# A New and Highly Sensitive Serum Mannoprotein Lateral Flow Assay for Point-of-Care Diagnosis of Invasive Aspergillosis (Tripathy Method)

**DOI:** 10.7759/cureus.26025

**Published:** 2022-06-17

**Authors:** Chandra P Chaturvedi, Zia Hashim, Naresh K Tripathy

**Affiliations:** 1 Department of Hematology, Stem Cell Research Center, Sanjay Gandhi Postgraduate Institute of Medical Sciences, Lucknow, IND; 2 Department of Pulmonary Medicine, Sanjay Gandhi Postgraduate Institute of Medical Sciences, Lucknow, IND; 3 Department of Hematology, Sanjay Gandhi Postgraduate Institute of Medical Sciences, Lucknow, IND

**Keywords:** sensitivity, point-of-care test, asplfd method, tripathy method, serum mannoprotein lateral flow assay

## Abstract

Background and objectives

The mannoprotein lateral flow assay (MP-LFA) or Aspergillus-specific lateral flow device (AspLFD) is a novel rapid test for point-of-care diagnosis (PoC) of invasive aspergillosis (IA), but its routine clinical application is hampered due to low sensitivity (S_n_) of the assay in serum. Therefore, this study aimed to develop a new method to enhance the S_n _of the serum MP-LFA.

Methodology

In the new method (Tripathy method), we used direct heating of the serum without any dilution at 120^°^C for 15 minutes to purify the mannoprotein (MP) antigen of the Aspergillus. The MP-enriched serum supernatant obtained after centrifugation was loaded in an LFD cassette, and the results were read after 20 minutes using a digital cube reader. In parallel to our new method, AspLFD was performed according to the manufacturer’s instructions. The diagnostic performance of the two methods was evaluated using paired sera of true IA patients (IA, n=18) and healthy subjects (controls, n=20). The positivity of the two methods was also evaluated in the sera of leukemia patients with possible/probable IA (possible/probable IA; n=23).

Results

The Tripathy method had a significantly higher sensitivity (88.9% versus 55.5%; p<0.05) and diagnostic odds ratio (72.0 versus 23.7) than the standard AspLFD method. In receiver operating characteristic curve analysis for differentiation between IA patients and controls, although the Tripathy method (area under curve; AUC: 0.894, p<0.001) and AspLFD method (AUC: 0.753, p<0.001) were significantly associated with IA, the AUC of the Tripathy method was significantly higher than that of the AspLFD method (0.894 versus 0.753; p<0.05). In the sera of possible/probable IA, MP-LFA by the Tripathy method had a significantly higher rate of positivity than the AspLFD method (39.0% versus 21.7%; p<0.05).

Conclusion

Our data show that the Tripathy method is a highly sensitive method of MP-LFA for the PoC diagnosis of IA in clinical settings.

## Introduction

Invasive aspergillosis (IA) is a life-threatening fungal disease caused by Aspergillus spp. that globally affects various immunocompromised patients, including those with hematological malignancies on chemotherapy, immunosuppressive/immunomodulatory medications, neutropenia, stem cell, and organ transplantation, as well as immunocompetent and non-neutropenic patients with lung disease and viral or bacterial pulmonary infections [1-4]. The outcome of the disease is poor, with mortality up to 90%, and the major cause behind such high mortality in IA is missed or delayed diagnosis of the disease. Hence, an early diagnosis of IA is crucial for effective anti-fungal treatment and improved outcomes of the disease [5, 6].

Since clinical and radiological signs of IA are often nonspecific, mycological diagnostic tests are required to substantiate diagnosis in almost all clinical settings [7, 8]. The mycological diagnostic tools currently used in routine clinical laboratories for definitive diagnosis of IA include fungal culture, direct microscopy, and detection of galactomannan (GM) and/or Aspergillus polymerase chain reaction (PCR) assays [8]. However, these tools have several practical limitations in terms of low sensitivity and/or large turnaround time. The sensitivity of fungal culture or microscopy of samples lies in the range of 20% to 50% [9, 10]. The Aspergillus biomarker-based GM assay has better sensitivity than microscopy and culture but it requires large numbers of samples to run on 96-well plates and involves time-consuming incubation processes [11]. Moreover, to reduce test cost, it is run in batches once or twice weekly, which increases the turnaround time and further delays the diagnosis. Aspergillus PCR is generally not available in routine laboratories for IA diagnosis due to a lack of proper standardization and validation. Moreover, its diagnostic performance has significant variation across studies and settings, with particularly poor performance in blood for diagnosing IA [12-14]. Thus, there is a compelling need for a point-of-care (PoC) diagnostic tool for the rapid and sensitive detection of IA in critical patients admitted to the hospital and intensive care unit (ICU).

Recently, a mannoprotein lateral flow assay (MP-LFA) has been commercially launched as a novel CE-marked Aspergillus-specific lateral flow device (AspLFD) assay for individual patient-based PoC diagnostic testing of IA in approximately 30 minutes with minimal laboratory infrastructure and technical requirement (OLM Diagnostics, Newcastle Upon Tyne, United Kingdom; http://olmdiagnostics.com). This is a simple and cost-effective adjunct test for rapid interim diagnosis of IA in hospitalized patients. It is based upon the immunochromatographic lateral flow principle using a mouse monoclonal antibody JF5 that detects an early germ tube-specific extracellular mannoprotein (MP) antigen secreted by Aspergillus spp. during active growth in host tissues [15]. AspLFD is recommended by the manufacturer for the detection of Aspergillus antigens in serum and bronchoalveolar (BAL) fluid. While standardizing this test for routine clinical diagnostic use, we observed its excellent diagnostic performance on BAL but limited diagnostic value on serum, which is the most commonly used non-invasive source of test samples for diagnostic testing. Several serum samples of even Aspergillus culture-positive patients showed either low positive or negative results.

We hypothesized that the sample pre-treatment used in the commercial AspLFD method affects the actual concentration of the target antigen in the serum either due to excessive (two times) dilution and/or ethylenediaminetetraacetic acid (EDTA) present in the dilution buffer. This prompted us to develop a new method without dilution of serum to augment the diagnostic value of AspLFD, and we have designated this new method of MP-LFA as "Tripathy Method." Here, we aimed to present the performance of the Tripathy method in comparison to the manufacturer’s AspLFD method for the diagnosis of IA using sera from true IA patients and healthy control subjects.

## Materials and methods

Subjects

Sera of hematology patients and COVID-19-associated pulmonary aspergillosis (CAPA) patients received in the microbial hematology laboratory of the Department of Hematology at Sanjay Gandhi Postgraduate Institute of Medical Sciences (SGPGIMS) from September 2020 to March 2022 for routine GM testing were used in this study. The patients with hematological diseases (n=13) and CAPA patients (n=5) underwent bronchoscopy and computed tomography (CT) of the chest at SGPGIMS. Patients who had Aspergillus-positive bronchoalveolar lavage fluid (BAL) by mycological culture, GM-positive BAL, and serum and radiological signs of IA were included as true IA patients (IA patients; n=18(13+5)). Acute leukemia (AL) patients (n=23) with clinical symptoms (e.g., antibiotic-resistant fever, dry cough, or pleuritic chest pain) and radiological signs of IA and serum positive or negative for GM were included as possible/probable IA patients (possible/probable IA). In addition, paired sera of age/sex-matched healthy subjects (n=20) were used as no IA subjects (controls) in the study. The diagnosis and categorization of IA in patients with hematological diseases were performed as per the European Organization for Research and Treatment of Cancer and the Mycoses Study Group (EORTC/MSG) criteria [16], while in CAPA patients, IA was defined as per the practice guidelines for CAPA criteria recently published by our group [17]. GM testing was performed as a routine investigation using the PlatelliaTM assay (Bio-Rad, France), and the GM index threshold was taken as >0.5. The MP-LFA by the Tripathy method and AspLFD method was performed in paired serum samples of patients and controls aseptically stored frozen at -80°C. The study was approved by the Institutional Ethics Committee of the SGPGIMS (IEC Reference Code # 2021-190-IMP-EXP-41).

Tripathy method of MP-LFA

We optimized the purification of low abundant and thermostable mannoprotein of Aspergillus by heating the undiluted sera and sera diluted 1:2, 1:4, 2:1, and 4:1 with EDTA buffer at 100°C and 120°C for 5, 10, 15 and 20 minutes. The Aspergillus antigen enriched supernatant generated by heating the serum at 120°C for 15 minutes yielded the best results within 20 minutes in repeated testing. This optimized procedure is described below in detail.

**Figure 1:**
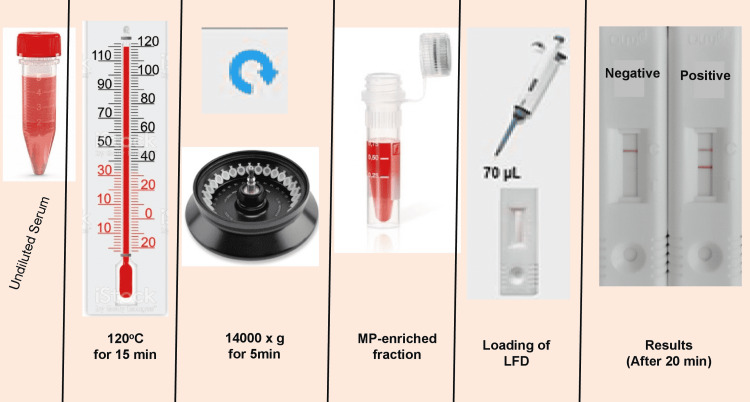
Main steps of Tripathy method of MP-LFA This figure is author’s own creation and design. AspLFD: Aspergillus-specific lateral flow device, MP-LFA: mannoprotein lateral flow assay

After thawing and vortexing, 350 µL of serum was transferred to a sterile 1.5 mL screw-capped tube and directly heated in a dry heat block at 120°C for 15 min. The heated serum was immediately centrifuged at 14000 × g for 5 minutes, and 70 µL of the supernatant was removed using a micropipette and carefully added to the sample port of the LFD cassette at ambient temperature. The LFD cassette was incubated at room temperature for 20 minutes to allow the supernatant to migrate to the result port and form the control line and Aspergillus-specific test line following immunoreaction. The results were read using a digital cube reader and expressed as negative (-), low positive (+) or positive (+++). The numeric cut-off value of MP was 35ng/mL [15]. A positive result was indicated by the appearance of both control and test lines, while a negative result showed only the control line. A test result, whether it was low positive (+) or strong positive (+++), was considered positive. If no control line was observed, the test result was considered invalid, and it was repeated on the same sample. A graphical sketch of the Tripathy method is shown in Figure 1.

AspLFD method of MP-LFA

**Figure 2:**
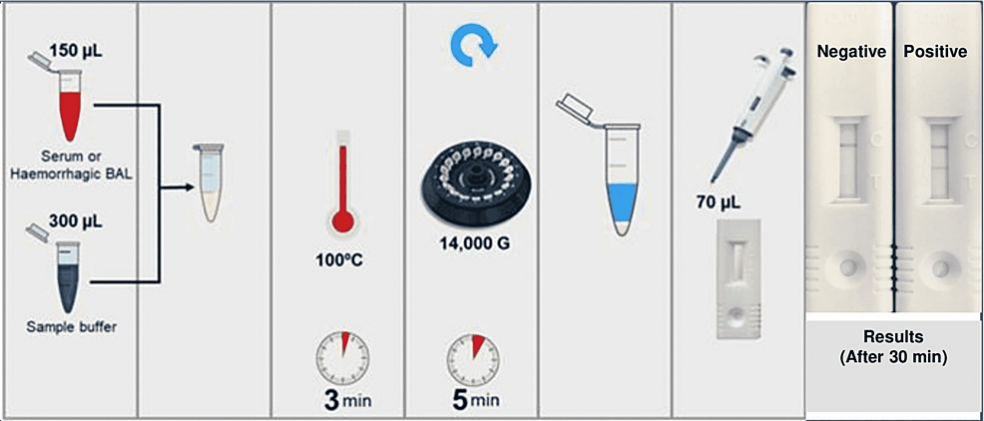
Main Steps of AspLFD method of MP-LFA This figure is modified from the AspLFD method published in Handbook HB002-V5, OLM Diagnostics (http://olmdiagnostics.com) with due permission of the company. AspLFD: Aspergillus-specific lateral flow device, MP-LFA: mannoprotein lateral flow assay

The AspLFD assay (OLM Diagnostics) was performed in accordance with the manufacturer’s protocol. Briefly, after thawing, vortexing, and centrifugation at 14,000 × g for 1 minute, 150 µL of serum was transferred to a sterile 1.5 mL screw-capped tube, and 300 µL of sample buffer was added to it (150 µL serum + 300 µL sample buffer; dilution 1:2). The tube was now heated in a dry heat block at 100°C for three minutes. This heated mixture was centrifuged at 14000 × g for five minutes, and 70 µL of supernatant was removed using a micropipette and carefully added to the sample port of the LFD cassette. The LFD cassette was incubated at room temperature for 30 minutes to allow the supernatant to migrate to the result port and form control (C) and Aspergillus-specific test (T) lines. The results were read using a digital cube reader and expressed as negative or positive as described above for the Tripathy method. A graphical sketch of Tripathy method is shown in Figure 2.

Statistical analysis

For statistical analysis, serum samples of IA patients and healthy subjects (controls) were taken as true positive and true negative samples, respectively. The sensitivity (Sn), specificity (Sp), positive predictive value (PPV), and negative predictive value (NPV) were calculated using these positive and negative groups. The performance of the two methods (including 95% confidence intervals (CI)) was compared for differentiation between IA patients and controls using descriptive analysis and Fisher’s exact test. The Sn, Sp, PPV, NPV, and diagnostic odds ratio (DOR) were computed and reported with a 95% CI taking the prevalence of IA as 32%. The diagnostic odds ratio (DOR) and its confidence interval were calculated manually using method of Glas et al, 2003. Receiver operating characteristic (ROC) curve analyses and area under curve (AUC) values were presented, including 95% CI for the outcomes of IA patients versus the control group. All statistical analyses were performed using SPSS Statistics v. 23 (IBM Corp., Armonk, NY), MedCalc for Windows, version 20.1 (MedCalc Software, Ostend, Belgium). A two-sided p-value of <0.5 was considered to be significant.

## Results

A total of 18 paired serum samples from IA patients and 20 age- and sex-matched healthy subjects (controls) were included in the study to analyze the diagnostic performance of the Tripathy and AspLFD methods. In addition, 23 paired sera from possible/probable IA patients were included for testing the rate of positivity of the MP-LFA test by the two methods. The demographic characteristics and underlying diseases of the IA patients and possible/probable IA patients are shown in Table 1.

**Table 1:** Demographic characteristics of IA (n=18) and possible/probable IA patients (n=23) IA: invasive aspergillosis, ALL: acute lymphoblastic leukemia, AML: acute myeloid leukemia, MDS: myelodysplastic syndrome, CLL: chronic lymphoblastic leukemia, MM: multiple myeloma, CAPA: COVID-19-associated pulmonary aspergillosis, APML: acute promyelocytic leukemia, HCL: hairy cell leukemia

Characteristics	IA Patients	Possible IA Patients
Number	18	23
Females/Males	11/7	13/10
Age (Years) : median (range)	38 (21-65)	42 (23-58)
Underlying disease		
ALL	05	09
AML	04	09
AML with Sepsis	None	01
MDS	02	None
CLL	01	02
MM	01	None
CAPA	05	None
APML	None	01
HCL	None	01

**Figure 3:**
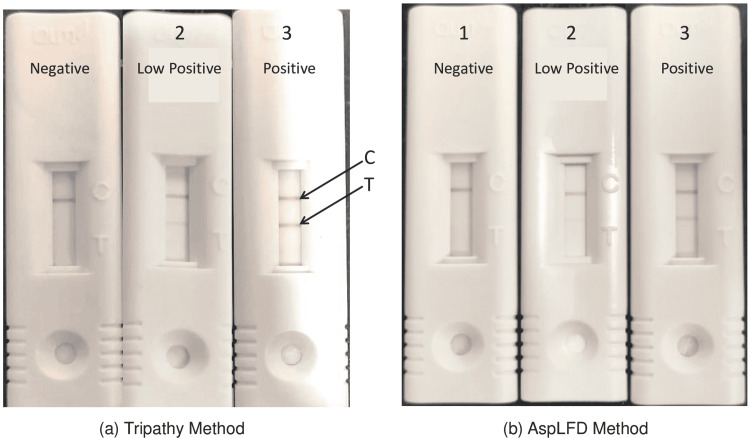
Results of MP-LFA showing representative (1) negative, (2) low positive, and (3) positive results in paired serum samples of IA patients and controls by the (a) Tripathy method and (b) AspLFD method The control line (C) is at the top, while a test (T) line of the assay is located below the control line. The test is interpreted as "positive" if both control and test lines are observed or "negative" if only the control line is observed. The intensity of the test line in the assay is directly proportional to the Aspergillus antigen in the serum samples. The Tripathy method, compared to the AspLFD method, has a visually higher intensity of test lines in the paired low positive and positive sera of IA patients. MP-LFA: mannoprotein lateral flow assay, AspLFD: Aspergillus-specific lateral flow device, C: control line, T: test line

The Tripathy method of MP-LFA was developed by varying the heating temperature and time of heat treatment of diluted and undiluted serum of the same patients. The heat treatment of sera at 120°C for 15 minutes was observed to be optimal for the assay. The MP-LFA results of the supernatants of highly (1:2 or 1:4) diluted sera revealed low intensity or no test line, those of mildly (2:1 or 4:1) diluted sera revealed a high-intensity test line whereas the best intensity of the test line was observed for undiluted sera (data not shown). Thus the purification of the Aspergillus antigen by direct heating of undiluted serum at 120°C for 15 minutes was used as the Tripathy method of MP-LFA assay. The method stated by the manufacturer (OLM Diagnostics) was used as the AspLFD method. The representative negative, low positive, and positive results of MP-LFA obtained using paired sera of IA patients and controls by the two methods are shown in Figure 3. The test line in the results of the low positive and positive serum samples of IA patients is visually clearer and brighter with the Tripathy method than with the AspLFD method as shown in Figure 3.

**Figure 4:**
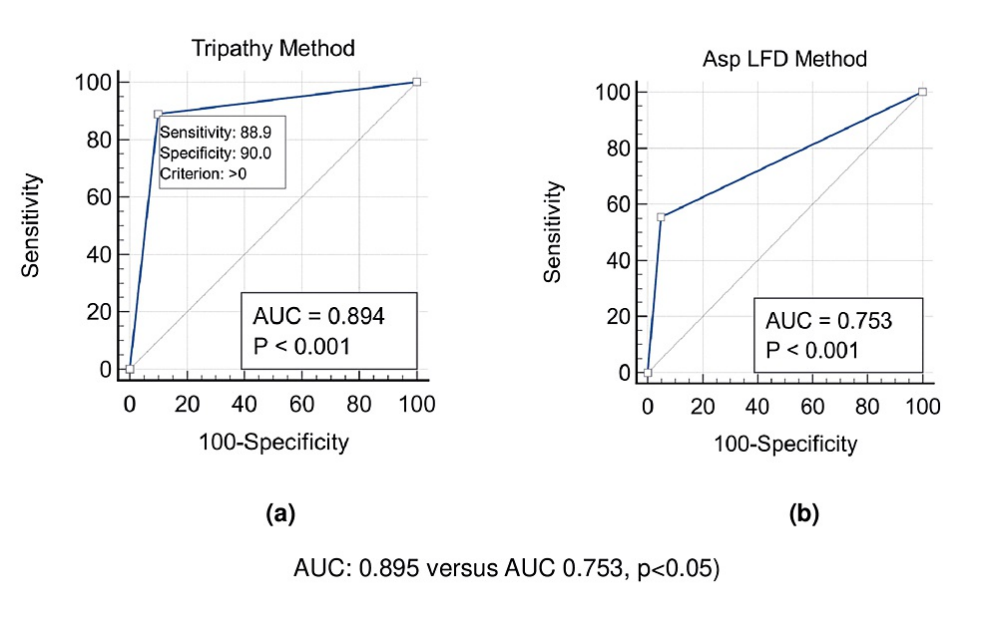
Receiver operating characteristic (ROC) curve analysis (95% confidence interval) of serum MP-LFA for diagnosing IA versus controls (a) Tripathy method, AUC: 0.894, p<0.001; and (b) AspLFD method, AUC: 0.753, p<0.001. Difference between AUC of Tripathy method and AspLFD method: 0.141; p<0.05. ROC: receiver operating characteristic, AspLFD: Aspergillus-specific lateral flow device, AUC: area under curve, MP-LFA: mannoprotein lateral flow assay

The Sn of the Tripathy method was significantly higher than that of the AspLFD method (88.9% (95% CI: 65.3 - 98.6) versus 55.5% (95% CI: (30.7-78.5); p<0.05). The DOR of the Tripathy method was 72.0 (95% CI: 9.7-402.8), while that of the AspLFD method was 23.7 (95% CI: 3.3-268.5). However, the two methods had a comparable Sp (90.0% (95% CI: 68.3- 98.8) versus 95.0% (95% CI: 75.1-99.8), p=not significant (NS)), PPV (80.7% (95% CI: 52.6-94.0) versus 83.9 (95% CI: 42.5-97.4); p=NS), and NPV (94.5% (95% CI: 82.2- 98.5) versus 70.3 (95% CI: 72.8-88.5); p=NS) (Table 2). In ROC curve analysis for differentiation between IA patients and controls, although the Tripathy method (AUC: 0.894 (95% CI: 0.752- 0.970), p<0.001) and the Asp LFD method (AUC: 0.753 (95% CI: 0.586-0.878), p<0.001) were significantly associated with IA, the AUC of the Tripathy method was significantly higher than that of the AspLFD method (0.894 (95% CI: 0.752- 0.970) versus (0.753 (95% CI: 0.586-0.878); p<0.05) (Figure 4). The two methods had similar MP-LFA results in IA sera with a high GM index. However, IA sera with a low or borderline GM index had higher positivity by the Tripathy method than by the AspLFD method (Table 3).

**Table 2:** Diagnostic performance parameters of serum MP-LFA of Tripathy method and AspLFD method in IA versus controls MP-LFA: mannoprotein lateral-flow assay, AspLFD: Aspergillus-specific lateral flow device, Sn: sensitivity, Sp: specificity, PPV: positive predictive value, NPV: negative predictive value, DOR: diagnostic odds ratio, CI: confidence interval, NS: not significant

Test Method	S_n_ (95% CI)	S_p_ (95% CI)	PPV (95% CI)	NPV (95% CI)	DOR (95% CI)	Test Positivity in Possible/Probable IA
Tripathy Method	88.9% (65.3 - 98.6)	90.0% (68.3- 98.8)	80.7% (52.6-94.0)	94.5% (82.2- 98.5)	72.0 (9.7-402.8)	39.0%
AspLFD Method	55.5% (30.7-78.5)	95.0% (75.1-99.8)	83.9% (42.5-97.4)	70.3% (72.8-88.5)	23.7 (3.3-268.5)	21.7%
p-value	<0.05	NS (>0.05)	NS	NS	Not Applicable	<0.05

**Table 3:** MP-LFA results by Tripathy and AspLFD methods in IA patients (n=18) MP-LFA: Mannoprotein lateral-flow assay, AspLFD: Aspergillus-specific lateral flow device, IA: invasive aspergillosis, ALL: acute lymphoblastic leukemia, AML: acute myeloid leukemia, MDS: myelodysplastic syndrome, CLL: chronic lymphoblastic leukemia, MM: multiple myeloma, CAPA: COVID-19-associated pulmonary aspergillosis

Patient Code	Disease	CT Findings	BAL Culture	BAL GM	Serum GM	Results	
						Tripathy Method	AspLFD Method
IA-1	ALL	Nodules	A. fumigatus	2.18	1.02	Positive	Negative
IA-2	ALL	Nodules and ground glass opacities	A. fumigatus	5.03	0.74	Positive	Positive
IA-3	ALL	Nodules	A. fumigatus	3.12	0.72	Positive	Positive
IA-4	ALL	Halo sign	A. niger	0.98	4.61	Positive	Positive
IA-5	ALL	Cavitary lesions	A. fumigatus	1.54	1.27	Positive	Positive
IA-6	AML	Nodular lesions	A. flavus	1.32	0.60	Positive	Positive
IA-7	AML	Nodules and ground glass opacities	A. niger	0.82	0.54	Positive	Negative
IA-8	AML	Nodules and halo sign	A. fumigatus	6.33	0.64	Positive	Negative
IA-9	AML	Nodules and halo sign	A. flavus	2.09	0.52	Positive	Negative
IA-10	CLL	Nodular lesions	A. flavus	1.72	0.68	Negative	Negative
IA-11	MM	Air crescent sign	A. fumigatus	3.02	1.16	Positive	Positive
IA-12	MDS	Nodules and halo sign	A. fumigatus	3.19	1.34	Positive	Negative
IA-13	MDS	Nodules and halo sign	A. fumigatus	6.51	1.07	Positive	Positive
IA-14	CAPA	Ground glass opacities, pulmonary nodules	A. terreus	1.66	2.09	Positive	Positive
IA-15	CAPA	Cavitary lesion, ground glass opacities	A. niger	1.91	1.55	Positive	Positive
IA-16	CAPA	Ground glass opacities, and pulmonary nodules	A. fumigatus	2.73	3.25	Positive	Positive
IA-17	CAPA	Ground glass opacities, and pulmonary nodules	A. flavus	1.37	0.72	Negative	Negative
IA-18	CAPA	Pulmonary nodules with halo sign, and ground glass opacities	A. fumigatus	4.09	0.61	Positive	Negative

The application of the two methods in possible/probable IA patients showed that MP-LFA by the Tripathy method had a significantly higher rate of positivity than the AspLFD method (39.0% versus 21.7%; p<0.05) (Table 2). While the sera of possible/probable patients with a high index had comparable MP-LFA results by the two methods, the sera of several of these patients with a borderline or negative GM index were positive by the Tripathy method but negative by the AspLFD method (Table 4).

**Table 4:** Rate of positivity of MP-LFA by Tripathy method and AspLFD method in possible/probable IA; n=23). MP-LFA: Mannoprotein lateral-flow assay, AspLFD: Aspergillus-specific lateral flow device, ALL: acute lymphoblastic leukemia, AML: acute myeloid leukemia, MDS: myelodysplastic syndrome, CLL: chronic lymphoblastic leukemia, APML: acute promyelocytic leukemia, HCL: hairy cell leukemia

Patient Code	Disease	CT Findings	Serum GM	MP-LFA Results	
				Tripathy Method	AspLFD Method
AL-1	ALL	Pleural effusion,	2.50	Positive	Positive
AL-2	AML	Not done	3.33	Positive	Positive
AL-3	APML	Not done	1.58	Negative	Negative
AL-4	AML	Focal airspace opacity	1.21	Negative	Negative
AL-5	HCL	Focal basilar airspace opacities	2.0	Positive	Positive
AL-6	ALL	Focal basilar airspace opacities	1.23	Positive	Positive
AL-7	AML	Not done	2.19	Negative	Negative
AL-8	AML	Diffuse bilateral interstitial opacities;	1.67	Negative	Negative
AL-9	CLL	Pleural effusion,	3.90	Positive	Positive
AL-10	AML with Sepsis	Peribronchial thickening	0.55	Positive	Negative
AL-11	ALL	Pleural effusion/thickening	0.52	Negative	Negative
AL-12	CLL	Lymphadenopathy	0.50	Negative	Negative
AL-13	ALL	Lymphadenopathy	0.52	Negative	Negative
AL-14	AML	Not done.	0.62	Positive	Negative
AL-15	ALL	Nodules with halo sign	0.17	Negative	Negative
AL-16	AML	Nodules with ground glass opacities	0.37	Negative	Negative
AL-17	AML	Nodular lesion	0.18	Negative	Negative
AL-18	ALL	Cavitary lesions	0.21	Negative	Negative
AL-19	AML	Ground-glass attenuation	0.17	Negative	Negative
AL-20	ALL	Bronchiectasis	0.31	Positive	Negative
AL-21	ALL	Consolidation	0.34	Positive	Negative
AL-22	ALL	Crazy-paving appearance	0.29	Negative	Negative
AL-23	AML	Nodular lesions with halo sign	0.27	Negative	Negative

## Discussion

AspLFD is an MP-LFA-based novel rapid test for the detection of Aspergillus antigen in serum and other body fluids, but its low Sn in serum has restricted its application as a PoC test for routine clinical diagnosis of IA. In the present study, we have developed a new method (the Tripathy method), which includes testing of the Aspergillus antigen enriched fraction obtained by direct heating of undiluted serum at 120°C. The Sn of this method was significantly higher than that of the AspLFD method. In addition, the Tripathy method also had a higher DOR and AUC than the AspLFD method, showing the diagnostic superiority of the former method over the latter. To our knowledge, Sn of 88.9% obtained in this study by the Tripathy method is the highest Sn of MP-LFA achieved in serum testing to date using the current CE-marked or prototype LFD by any method, and the Tripathy method represents the first highly sensitive method of MP-LFA to facilitate the application of this test for PoC diagnosis of IA in clinical settings.

Human serum contains high contents of various proteins, such as albumin, immunoglobulins, and immune complexes that interfere with the detection of low-abundant serum components like Aspergillus-derived MP antigen. Moreover, excessive protein contents make the serum viscous hindering its capillary action, which is crucial for LFA-based assays like MP-LFA. Hence, pre-fractionation of native contents of the target antigen of Aspergillus from abundant serum proteins is crucial for optimal capillary action of the antigen-enriched serum fraction for movement in the LFD cassette and for proper detection of the antigen by mAb JF5 to obtain accurate MP-LFA results. Most serum proteins are thermolabile and can be removed by heat precipitation to purify low-abundance thermostable proteins and other components of serum [18, 19]. In this study, we directly heated neat (undiluted) sera to precipitate the unnecessary serum proteins to obtain MP antigen-enriched serum supernatant with optimal capillary action. By varying the temperature and the heat treatment time, the heating of serum at 120°C for 15 min was found to be optimal for the assay, and this optimized condition was used in this study as the Tripathy method. Thus, serum supernatant obtained by the Tripathy method contains actual contents of the Aspergillus antigen present in the blood of the patients to offer accurate and sensitive results. In contrast, the AspLFD method uses two times dilution of patient sera with a sample buffer containing EDTA, and this excessive dilution may alter the actual levels of MP in the serum, resulting in low sensitivity of this method. EDTA has been reported to have fungicide activity and can destroy fungal cell wall contents, including MP [20, 21]. Thus, the use of EDTA in the sample pre-treatment of the AspLFD method may also be an important factor behind the low sensitivity of this method. In addition, the AspLFD method includes heating at 100°C for three minutes, which may be insufficient to remove several of the interfering serum proteins, and thus may be responsible for the low sensitivity of the method. The MP-LFA results of low positive sera also had a clearly visible test-line in the Tripathy method due to the higher sensitivity of this method while such sera had no visually clear test line in the AspLFD method. This shows that the results of the Tripathy method can be visually interpreted without a digital cube reader.

We observed a lower intensity test line in the MP-LFA results of highly diluted sera than mildly diluted sera of the same patients and the test line of the highest intensity in the results of the undiluted sera of the patients during the optimization of the Tripathy method. Similarly, two studies on prototype LFD have reported quite different Sn and Sp of the MP-LFA due to differences in the dilution of sera with EDTA buffer. One study using highly diluted (1:2) serum reported Sn and Sp as 40% and 86.8%, respectively [22]. The other study using mildly diluted (2.7: 1) diluted serum had Sn and Sp as 81.8% and 84.7%, respectively [23]. Our observation and results of these two studies together suggest that pre-treatment of serum with EDTA buffer may affect the sensitivity of MP-LFA.

A major difference in the diagnostic performance of the two methods lies in the IA patients having low or borderline serum levels of GM. The Tripathy method as compared to AspLFD method had higher positivity in the sera of such patients and thus it can better detect early disease. Similarly, sera of the possible/probable patients with borderline or negative GM index were positive by the Tripathy method but negative by the AspLFD method. This shows that MP is the earliest antigen secreted by Aspergillus during active hyphal growth when no detectable levels of GM are present in the patient serum. Corroborating our observation, an animal study has reported an earlier positivity of MP-LFA (day 3) than 1→3)-β-D-glucan and GM (Day 5) [24].

The newly formatted and CE-marked AspLFD used in this study was launched by OLM Diagnostics in 2018 [25]. Since then, only four clinical studies have reported its diagnostic performance in serum. One study in chronic pulmonary aspergillosis reported a Sn of 62%, which was comparable to the Sn of the AspLFD method but lower than that of the Tripathy method of the present study [26]. The remaining three studies in various hematologic patients with possible/probable IA had very low Sn ranging from 9% to 18% only [27-29]. Similarly, a recent meta-analysis of seven studies published between 2008 and March 2015 on the prototype LFD reported pooled Sn for sera of proven/probable IA cases as 68%, which is also comparable to the AspLFD method but lower than that of the Tripathy method of the present study [30]. In addition, Sp, NPV, PPV of Tripathy method were either comparable or superior to the AspLFD method but DOR (72.0 [9.7-402.8]) of the Tripathy method was substantially higher than the AspLFD method reported in these studies using CE-Marked AspLFD or prototype LFD (26-30). Thus, the Tripathy method with Sn 88.9% and DOR 72,0 (9.7-402.8) may be a highly sensitive and accurate method to serve as a potential PoC test for the diagnosis of IA. A major limitation of this study is its small sample size and hence the results obtained need to be validated by large-sized studies.

## Conclusions

We have developed a highly sensitive method (Tripathy method) of MP-LFA that has the following three major advantages over the commercial AspLFD method currently being used for CE-marked LFD: Firstly, its supernatant generated by direct heating of undiluted serum contains actual contents of the Aspergillus antigen present in the blood of the patients to offer more accurate results; secondly, its results have clearly visible test-line of sufficient intensity in all positive sera including low positive ones showing that this method can enable visually interpretation of the results without a digital cube reader; and thirdly, this method can better detect an early disease when GM levels are low or undetectable in the sera of IA patients. Further studies, including multicenter studies with sufficient sample size, are needed to validate the diagnostic performance of the Tripathy method of MP-LFA in the serum of patients with possible/probable IA and control patients with no IA for the recommendation of this method as a PoC test for clinical use in the routine diagnosis of IA.
